# Supplementary figures and images for: Endothelial Semaphorin 3fb regulates Vegf pathway-mediated angiogenic sprouting
Source: PLoS Genet. 2021 Aug 23;17(8):e1009769. doi: 10.1371/journal.pgen.1009769 (PMC8412281; doi:10.1371/journal.pgen.1009769)

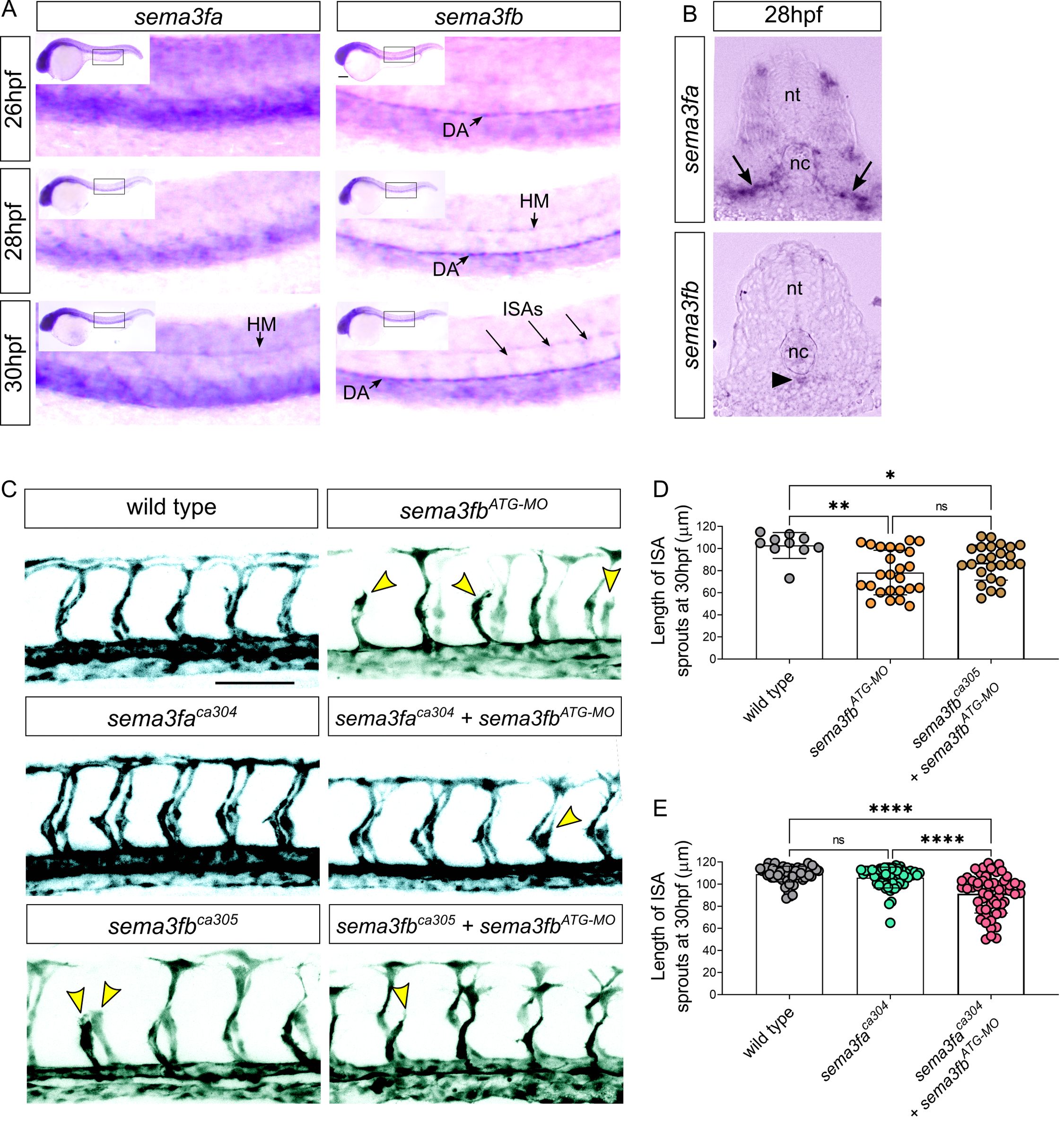

Supplement: S1 Fig — A) Lateral view of whole-mount ISH from 26-30hpf shows sema3fa expression in the ventral lateral somites and sema3fb expression in the dorsal aorta (DA) and intersegmental arteries (ISAs). HM: Horizontal Myoseptum. B) Expression of sema3fa and sema3fb in transverse sections of the trunk at 28hpf. sema3fa is expressed in ventral and lateral somite (arrows) while sema3fb is strongly expressed in the DA (arrowhead), Neural tube (nt), notochord (nc). C) Lateral confocal images of trunk vasculature (black) of 30hpf control wild type (WT), homozygous sema3faca304 and sema3fbca305 mutant embryos with and without injection of 1ng sema3fbATG-MO. Scale bar, 100 μm. n/N = number of embryos with angiogenic defects/Total number of embryos. D) Length of ISA sprouts in WT, sema3fbATG-MO morphants and sema3fb ca305 knockdown embryos with sema3fbATG-MO at 30 hpf; N = 1, WT = 2 embryos (10 ISAs, mean 105±7 μm), sema3fbATG-MO = 5 embryos (25 ISAs, mean of 78±20 μm), and sema3fbca305 + sema3fbATG-MO 5 embryos (25 ISAs, mean 87±15 μm), ****p<0.0001. E) Length of ISA sprouts in WT and sema3faca304 mutant and embryos with sema3fbATG-MO at 30 hpf; N = 2, 6 embryos per group: WT (30 ISAs, mean 109±7 μm), sema3faca304 (28 ISAs, mean of 106±9 μm), and sema3faca304 + sema3fbATG-MO (30 ISAs, mean 91±17 μm), ****p<0.0001. One-Way ANOVA Tukey’s multiple comparisons test. Error bars = ±SD. (TIF) [file pgen.1009769.s001.tif]

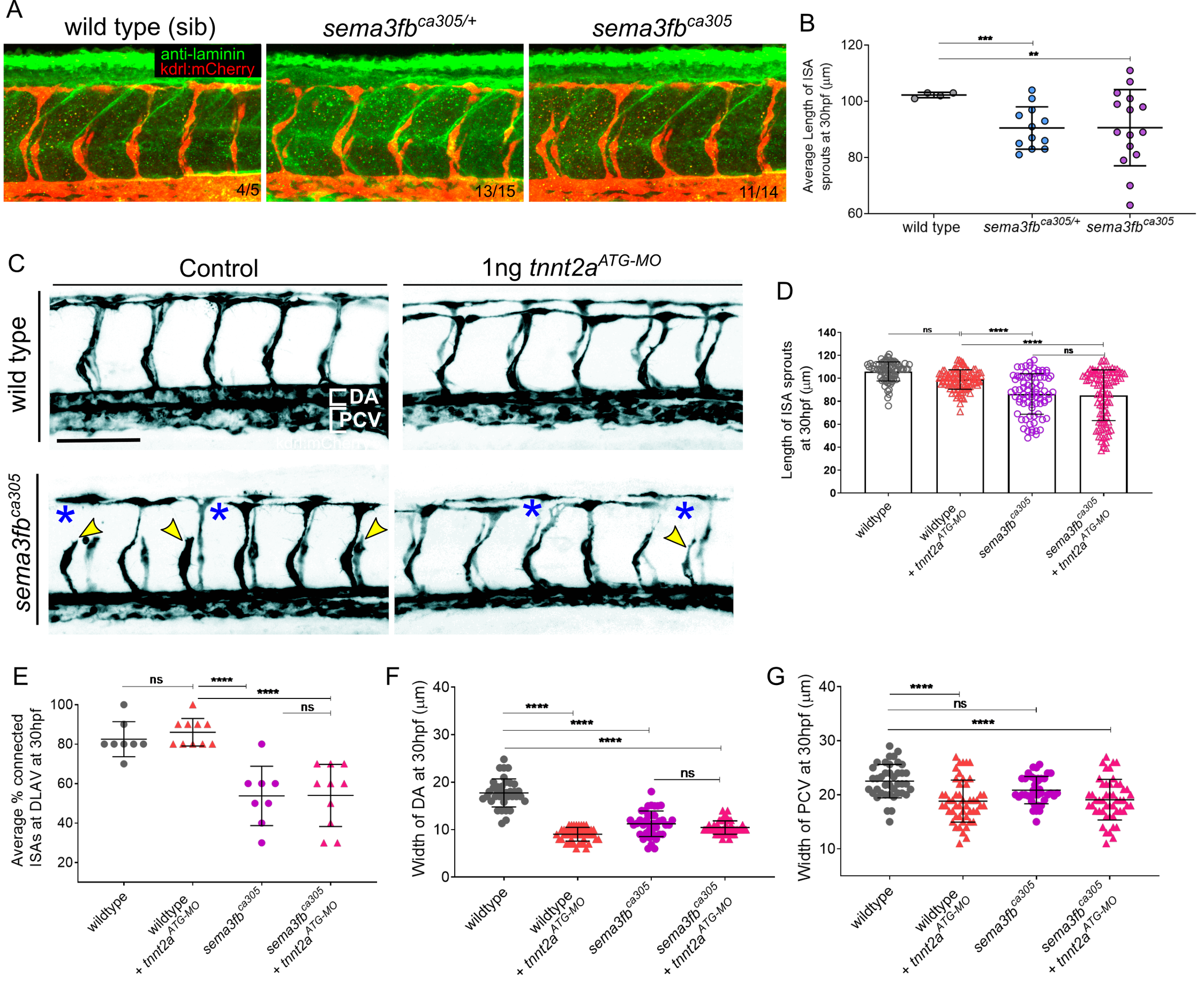

Supplement: S2 Fig — A) Confocal lateral images of laminin-stained embryos at 30hpf. Tg(kdrl:mCherry) endothelium (red) and laminin (green). Embryos derived from a heterozygous sema3fbca305/+ incross. B) Quantification of the length of ISA sprouts at 30hpf, N = 1: wild type (WT) (40 ISAs, 4 embryos, mean of 102±1 μm2), sema3fbca305/+ (120 ISAs, 12 embryos, mean of 90±8 μm2), and sema3fbca305 (150 ISAs, 15 embryos, mean of 90±14 μm2). C) Confocal lateral images of the trunk endothelium (black) in blood flow-stopped tnnt2ATG-MO injected wild type siblings (WT) and sema3fbca305 mutants. DLAV gaps (blue asterisks) and truncated ISAs sprouts (yellow arrowheads) are marked. Scale bar, 100μm. D) Length of ISA sprouts at 30 hpf, N = 3: WT (80 ISAs, 8 embryos, mean length of 106±3 μm), WT + tnnt2aATG-MO (100 ISAs, 10 embryos, mean 98±8.4 μm), sema3fbca305 (80 ISAs, 8 embryos, mean 86±17 μm), and sema3fbca305 + tnnt2aATG-MO (100 ISAs, 10 embryos, mean 85±22 μm). E) Percentage of ISAs connected at DLAV at 30 hpf, N = 3: WT (mean 82±9% connected), WT + tnnt2aATG-MO (mean 86±7% connected), sema3fbca305 (80 ISAs, 8 embryos, mean 54±15% connected), and sema3fbca305 + tnnt2aATG-MO (mean 54±16% connected). F) Quantification of width of DA in 30 hpf embryos, N = 3: WT (8 embryos, 5 measurements per embryo/n = 40 total, mean width of 18±3 μm), WT + tnnt2aATG-MO (10 embryos, 5 measurements per embryo/n = 50 total, mean 9±1 μm), sema3fbca305 (8 embryos, 5 measurements per embryo/n = 40 total, mean 11±3 μm), and sema3fbca305 + tnnt2aATG-MO (10 embryos, 5 measurements per embryo/n = 50 total, mean 10±1 μm). G) Quantification of width of PCV in 30 hpf embryos, N = 3: WT (n = 40, mean width of 22±3 μm), WT + tnnt2aATG-MO (n = 50, mean 19±4 μm), sema3fbca305 (n = 40, mean 20±3 μm), and sema3fbca305 + tnnt2aATG-MO(n = 50, mean 19±4 μm). 2-Way ANOVA Tukey’s multiple comparisons test, **** means p = <0.001. Error bars = ±SD. (TIF) [file pgen.1009769.s002.tif]

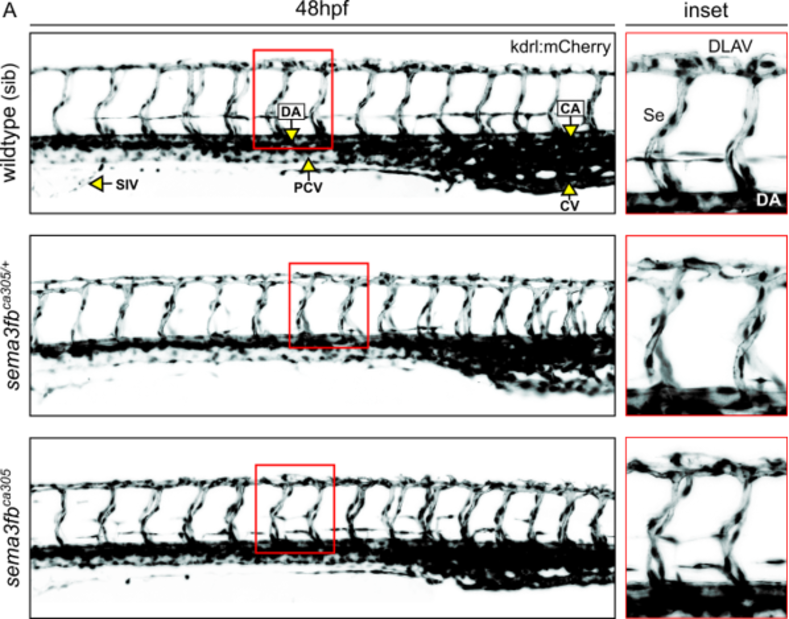

Supplement: S3 Fig — A) Representative Lateral confocal images of trunk vasculature (black) of 48 hpf control wild type (WT), heterozygous sema3fbca305/+ and homozygous sema3fbca305 mutant embryos with no obvious differences in vessel morphology or Segmental vessel (Se) connections. Scale bar, 100 μm. SIV = sub-intestinal vein (plexus), DA = Dorsal Aorta, PCV = Post Caudal Vein, CA = Caudal Artery, CV = Caudal Vein. N = 2, n: WT = 5, sema3faca305/+ = 5, and sema3fbca305 = 7. (TIF) [file pgen.1009769.s003.tif]

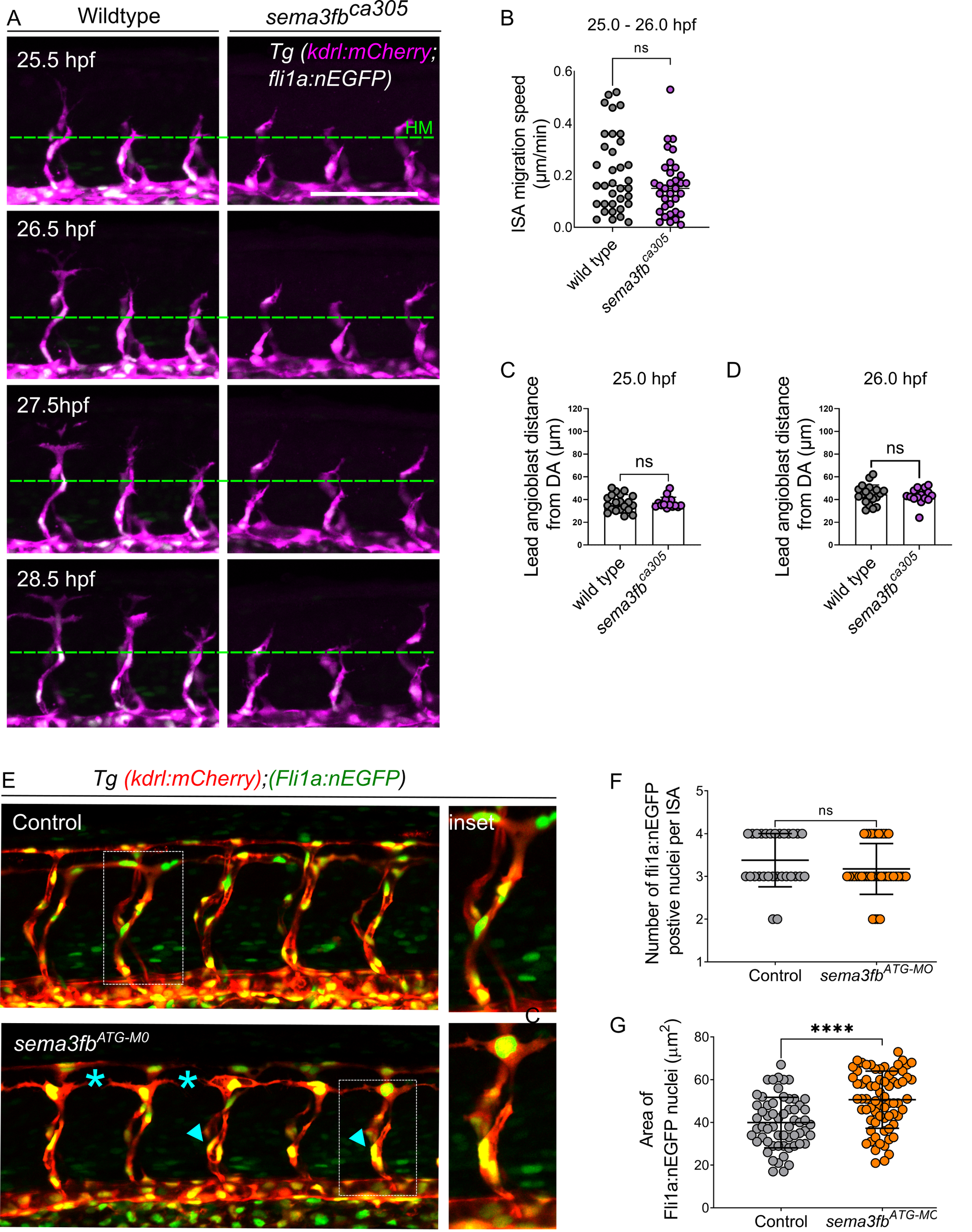

Supplement: S4 Fig — A) Lateral confocal timelapse images of time-lapse images in 25.5–28.5 hpf double transgenic Tg(kdrl:mCherry;fli1a:nEGFP) endothelial cells (magenta) and nuclei (white). The horizontal myoseptum (green dashed line) is noted to highlight ISA growth over time. Scale bar, 50 μm. B) During the 25–26 hpf interval there is no significant difference in speed between wild type and mutant embryos, see S2 Table for details. C) Lead angioblast mean distance from DA at 25 hpf: WT = 44.47±8.36 μm and sema3fbca305 = 43.14±6.84 μm, p = 0.609. D) Lead angioblast at 26 hpf mean distance from DA: WT = 55.12±14.06 μm and sema3fbca305 = 47.18±5.75 μm, p = 0.572. C-D) N = 1: WT = 4 embryos (20 ISAs) and sema3fbca305 = 3 embryos (15 ISAs), Unpaired t-test with Welch’s correction. E) Lateral confocal images of double transgenic Tg(kdrl:mCherry;fli1a:nEGFP) endothelium (red) and endothelial cell nuclei (green). DLAV gaps (blue asterisks) and truncated ISAs sprouts (white arrowheads) are noted. Embryos derived from a heterozygous sema3fbca305/+ incross. Scale bar, 100 μm. G) Quantification of the number of endothelial cell nuclei per ISAs in 30 hpf embryos, N = 2: WT (6 embryos, mean of 3 nuclei/ISA)), and sema3fbMO (7 embryos, mean of 3 nuclei /ISA). Unpaired t-test with Welch’s correction, p = 0.17. G) Quantification of the average area of endothelial cell nuclei per ISAs in 30 hpf embryos, N = 3: WT (60 ISAs, 6 embryos, mean of 42±16 μm2), and sema3fbMO (70 ISAs, 7 embryos, mean 61±19 μm2). Unpaired t-test with Welch’s correction, ****p<0.0001. Error bars = ±SD (TIF) [file pgen.1009769.s004.tif]

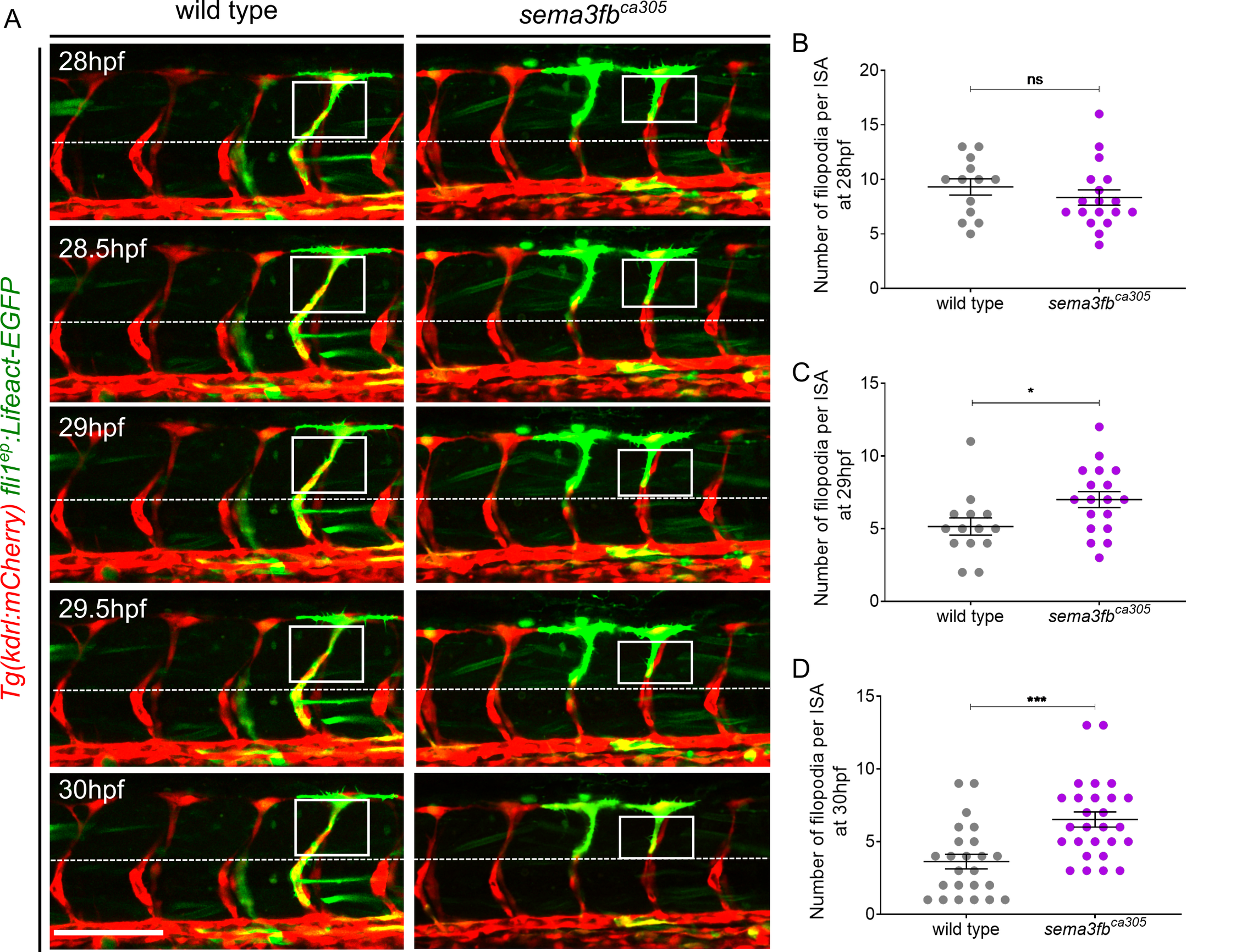

Supplement: S5 Fig — A) Representative still images of single-cell expression of fli1ep: Lifeact-EGFP (green) in ISA endothelial cells from 28-30hpf wildtype and sema3fbca305 embryo time-lapse imaging. A dashed white line represents the horizontal myoseptum and selected areas for filopodia counts are highlighted in white boxes. B) Quantification of number Lifeact-EGFP positive filopodia on ISA at 28hpf from embryos of the indicated genotypes. Unpaired t-test, p = 0.3566. C) Quantification of number Lifeact-EGFP positive filopodia on ISA at 29hpf from embryos of the indicated genotypes. Unpaired t-test, p = 0.0029. D) Quantification of number Lifeact-EGFP positive filopodia on ISA at 30hpf from embryos of the indicated genotypes. N = 3 for each quantification: WT (14 ISAs, 6 embryos, mean of 3 filopodia/ISA) and homozygous sema3fbca305 (18 ISAs, 7 embryos, mean of 8 filopodia/ISA). Unpaired t-test, p = 0.0002. (TIF) [file pgen.1009769.s005.tif]

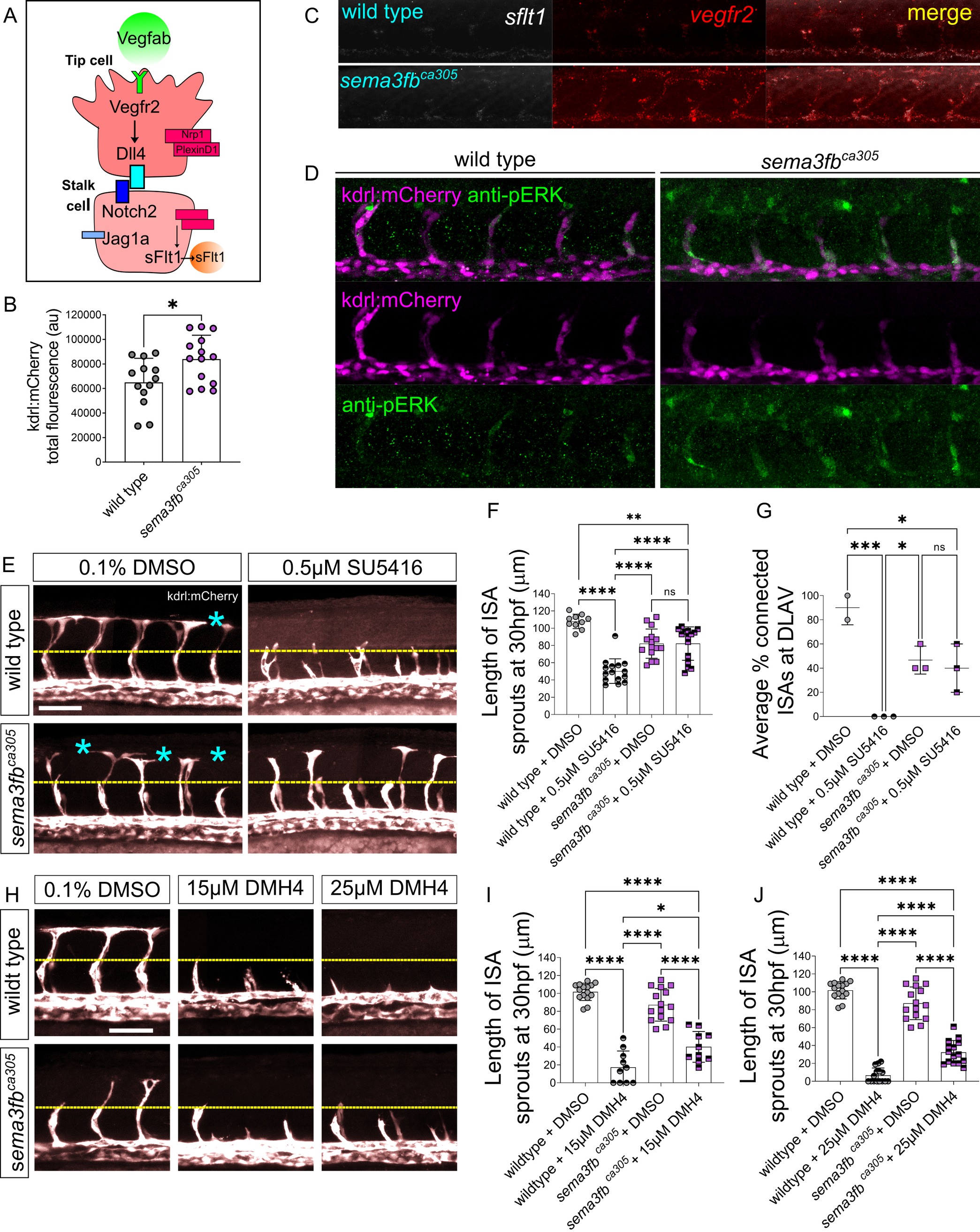

Supplement: S6 Fig — A) A model of signaling pathways that regulate angiogenic sprouting, highlighting key genes controlling tip and stalk cell identity. B) Quantification of Tg(kdrl:mCherry) transgene expression levels in wild type and sema3fbca305 ISAs at 30hpf. N = 3: wild type (n = 13 embryos, average of 6500 a.u.) and sema3fbca305 (n = 14 embryos, average of 8400 a.u.). T-test with Welches correction, *p = 0.0186, a.u. = arbitrary unit of intensity. C). B) Fluorescent HCR in situ for vegfr2 and sflt1 RNA transcripts in whole-mount wild type and embryos sema3fbca305 embryos fixed at 30 hpf. D) Whole-mount Immunostaining for phosphoERK (pERK) in WT and sema3fbca305 embryos fixed at 30 hpf. E) Lateral confocal images of the trunk vasculature Tg(kdrl:mCherry) (white) in embryos treated with 0.5 μM SU5416 from 20hpf-30hpf. DLAV gaps (blue asterisks) and ISA truncated sprouts (yellow dashed line at the level of horizontal myoseptum are indicated. Scale bar, 100 μm. F) Quantification of ISA sprout length in 30 hpf embryos treated with 0.5 μM SU5416, N = 1: WT + DMSO (25 ISAs, 5 embryos, mean of 107±8 μm), WT + 0.5μM SU5416 (25 ISAs, 5 embryos, mean of 50±14 μm), sema3fbca305 + DMSO (30 ISAs, 6 embryos, mean of 82±17 μm), and sema3fbca305 +0.5μM SU5416 (30 ISAs, 6 embryos, mean of 82±19 μm). G) Percentage of ISA sprouts connected at DLAV in 30 hpf embryos treated with 0.5 μM SU5416, N = 1: WT + DMSO (25 ISA-DLAV, 5 embryos, mean 78% of ISA-DLAV/embryo), WT + 0.5 μM SU5416 (25 ISA-DLAV, 5 embryos, mean of 78%), sema3fbca305 + DMSO (30 ISA-DLAV, 6 embryos, mean of 51%), and sema3fbca305 + 0.5 μM SU5416 (30 ISA-DLAV, 6 embryos, mean of 82±19%). Error bars = ±SD. H) Lateral confocal images of the trunk vasculature Tg(kdrl:mCherry) (white) in embryos treated with low doses of DMH4 μM SU5416 from 20hpf-30hpf. ISA truncated sprouts (yellow dashed line at the level of horizontal myoseptum are indicated. Scale bar, 50 μm. I) Quantification of length of ISA sprouts in 30 hpf embryos treated with 15 [file pgen.1009769.s006.tif]
