# Supplementary material for: Endothelial Semaphorin 3fb regulates Vegf pathway-mediated angiogenic sprouting
Source: PLoS Genet. 2021 Aug 23;17(8):e1009769. doi: 10.1371/journal.pgen.1009769 (PMC8412281; doi:10.1371/journal.pgen.1009769)
Supplement: S2 Table — (DOCX) [file pgen.1009769.s008.docx]

**S2 Table. Angioblast migration distance and speed**
