# Supplementary material for: Endothelial Semaphorin 3fb regulates Vegf pathway-mediated angiogenic sprouting
Source: PLoS Genet. 2021 Aug 23;17(8):e1009769. doi: 10.1371/journal.pgen.1009769 (PMC8412281; doi:10.1371/journal.pgen.1009769)
Supplement: Attachment — Submitted filename: Watterston PLOS Genetics reply to reviewers v3.docx [file pgen.1009769.s011.docx]

#### Reply to reviewers for PGENETICS-D-21-00136

We thank the reviewers heartily for insightful and fair suggestions that have greatly improved the manuscript. We address the points below.

As an overview, data additions we have made to the manuscript as suggested by the reviewers include:

- Timelapse analysis of migration
- ISH analysis (HCR) of vegfr2 and sFlt1 for confirmation of upregulation
- Phospho-ERK staining
- A second VEGFR2 inhibitor
- Morpholino data when applied to sema3fb mutants
- Phenotype analysis at 48 hpf after migration is complete

Reviewer #1: In this manuscript Watterston and colleagues investigate the function of the sema3fb gene in zebrafish vascular development. Using a sema3fb mutant that is predicted to express a truncated transcript, they reveal that intersegmental artery elongation is stunted and filopodia persist for longer in heterozygous and homozygous mutant embryos. These observations are associated with an increase in gene expression of angiogenesis inducing and repressing factors. Finally, Watterston et. al. nicely demonstrates that disruption intersegmental artery elongation in sema3fb mutants is reversed by 0.2µM SU5416, suggesting that excessive Vegfr2 activity underpins this mutant defect. The study is well presented, and I am positive about this work, however there are number of remaining important questions that need to be addressed before publication.

(1-1) Is Vegfr2 signaling truly increased in sema3fb mutants?

At several points in the text, it is suggested that the observed phenotype is due to increased vegfr2 expression resulting in an assumed increase in Vegfr2 activity in sema3fb mutants. However, increased vegfr2 expression does not confirm increased Vegfr2 activity, especially when sflt1 expression is also increased. The suggestion of increased Vegfr2 activity in sema3fb mutants needs to be confirmed to support the author’s model. Others have used pErk immunostaining or Erk reporters to characterize Vegfr2 activity during intersegmental artery elongation. These would be important tools to use to confirm the author’s hypothesis that increased vegfr2 expression leads to increased Vegfr2 activity.

- This is an excellent point that both reviewers raised. We stained for pERK at 30 hpf, the relevant timepoint for our study. The data, now presented in Figure 4 show that the intensity of pERK staining within positive nuclei is significantly increased (Figure 4 D,F). This result is consistent with increased VEGF signaling in sprouting ISA angioblasts. Taken together with the other data we previously provided supporting increased VEGFR2 expression and rescue of the phenotype with VEGF inhibitors, multiple lines of evidence point to VEGF activation being the mechanistic consequence of loss of *sema3fb*.

(1-2) Could defects in vessel branching be due to a delay in sema3fb mutant development?

Many aspects of the observed phenotype of sema3fb heterozygous and homozygous mutant embryos could be due to an embryonic delay or transient disruption to vessel branching. For example, does vessel elongation fully recover at later time points? Importantly, can the authors confirm that sema3fb mutant do not experience an overall developmental delay by quantifying classic embryonic stage markers, such as head-trunk angle and embryo length.

- Developmental delay was considered during all experiments. Firstly, all embryos were collected within a 10-minute window to ensure tight staging. There is no visible difference in embryonic timing by normal staging, and additionally, until 26 hpf, no vascular delay. We show this using time lapse (25-29 hpf) that there is identical timing for initiation of sprout formation (25 hpf), but that during ISA migration, mutant angioblasts show significantly slower rates of migration from 26hpf through to 29 hpf (Figure 2 A-H). Thus, this new data pinpoints the exact window where mutants start to differ from wildtype vascular development.
- Like other angiogenic mutants with sprouting delay (isl2a, Lamont et al., DevBio 2016), recovery of the sema3fb phenotype occurs, likely through compensation by unknown genes and pathways. We focus here on the early period of time where the initial formation of sprouts is impaired but have added phenotypic data of their recovery by 48 hpf (Supplementary Figure 6).

(1-3) Are persistent filopodia in sema3fb mutant a consequence of delayed anastomosis?

The authors reveal that sema3fb mutants retain intersegmental artery filopodia for longer than wild type siblings. However, earlier in the manuscript they also show that sema3fb mutant intersegmental arteries often fail to connect with each other by anastomosis to form the DLAV. As this anastomosis event is responsible for repressing filopodia, it seems possible that the persistent filopodia observed in sema3fb mutants could be an indirect consequence of this delayed anastomosis rather than a specific effect of sema3fb on actin remodeling. To clarify this point, the authors should confirm that they only quantified filopodia numbers on intersegmental arteries in sema3fb mutants that connected to form the DLAV at the exact same time point as in wild type sibling embryos (and not in slightly delayed vessels). If not, it would be important to reanalyze the data to take this into account.

- We only looked at vessels undergoing anastomosis in this mosaic experiment. This is why we picked the upper portion of the ISA below the DLAV for measurements, as filipodia resolution occurs in this portion of the ISA. Figure 3 now includes n’s in the legend, and highlights that although in stage matched embryos, nearly all the WT vessels reach and anastomose at the DLAV. Since only a proportion of the ISAs are GFP positive, 14 of 30 ISAs in 6 embryos were used for quantitation. In mutants a smaller proportion of vessels reach and anastomose with wild type timing, but we used 18 of 35 ISAs from 7 embryos. GFP-positive mutant ISA cells show higher numbers of filipodia, suggesting a delay in turning off filopodial activity. The individual data points are shown in Supplementary Figure 4. We have now clarified the experiment in the results and discussion. Given several hour window over which we measured filopodia, the mutants should have caught up angiogenic delay as the vessels we measured were in the process of anastomosis.

(1-4) Is endothelial cell motility disrupted in sema3fb mutants?

The short intersegmental arteries observed are likely a consequence of reduced endothelial cell motility, but this is not tested. Likewise, changes to endothelial cell nuclei spacing and shape are also potentially a consequence of reduced endothelial cell movement. It would be important to image intersegmental artery elongation in live embryos and quantify endothelial cell motility to confirm the cause of these vessel defects.

- This is an excellent point that we address with new time lapse data in Figure 2 . The timelapse highlights differences in migration kinetics mutants. Sprout initiation appears normal at 25 hpf, but the migration of the lead angioblast is significantly slower during the 26-29 hpf period.

(1-5) Does sema3fb truly form part of a “feedback mechanism”?

At several points in the text the authors indicate that sema3fb forms part of a feedback mechanism that regulates Vegfr activity. This concept is interesting but is not directly tested. The authors should determine if sema3fb gene expression is regulated by Vegfr signaling using mutants or chemical inhibitors of the Vegf pathway. If Vegf induces sema3fb expression, which then feeds back to inhibit excessive Vegfr activity, this might provide evidence of a bona fide feedback loop.

- We removed wording to suggest this is a feedback loop as it is premature to suggest this. Instead, we suggest that autocrine Sema3fb secretion is an additional layer of control on the VEGF pathway.

(1-6) What is the role of sflt1 in sema3fb-mediated control of intersegmental artery elongation?

On line 286, the authors propose that “reducing excessive Vegfr2 activity in sema3fb mutants would be expected to decrease levels of sFlt1, thus allowing sprouting to occur”. This is somewhat counterintuitive as high Vegfr2 activity is considered to induce sprouting. Moreover, it is unclear in this model how endothelial cells can maintain high levels of Vegfr2 activity if sFlt1 levels are so high that they repress this Vegfr2 activity. To support this proposed model, the authors should determine by qPCR if sflt1 levels return to normal in sema3fb mutants upon treatment with 0.2µM SU5416 or other Vegfr2 inhibitors.

- This is an interesting point, and we agree it seems counterintuitive, but our data is very robust. We show strongly supportive data for the upregulation of VEGFR2 (qPCR, HCR, and transgenic fluorescence quantification). At a functional level, we have strong evidence for upregulated VEGF signaling being important for the phenotype, as reflected by both pERK upregulation and rescue of the sema3fb sprouting phenotype by VEGF inhibition (now supported by 2 inhibitors). Taken together, all the data strongly point to increased VEGF signaling as leading to the phenotype.
- What about sFlt1? We now add HCR data to show that sFlt1 is also upregulated (previously shown only by qPCR). Thus, both the activating and inhibitory molecules are upregulated at the mRNA level. Overall, sFlt1 does not appear have a strong effect. As we allude to in our discussion, the big unknown here is what is happening to protein levels. SFlt1 is a protein that sops up VEGF extracellularly. Even if there is more mRNA for sFlt1, we currently have no way to assess its secreted protein levels. We do not think that qPCR of sflt1 after VEGF inhibition is the right experiment, but we trust that we have provided enough supportive information to show the key role that VEGFR2 plays in the sema3fb phenotype.

Minor points:

1-7- It would be important to confirm the specificity of the sema3fb morpholino tool (as well as sema3fb haploinsufficiency) by injecting this morpholino into heterozygous and homozygous sema3fb mutant embryos to confirm that no additional phenotype is observed.

- We now show images and quantification of the morpholino on its own and in combination with sema3fb genetic mutants. There are no additional phenotypes. Morpholino data is also included in the appended cardiac manuscript, currently also submitted as an independent study to PLOS Genetics. We attach the manuscript here which has also been deposited in BioRXiv (doi: <https://doi.org/10.1101/2021.05.19.444704>, reference [39]) for the reviewers to assess the data.

1-8- SU5416 is not a specific inhibitor of Vegfr2 signaling and impacts many unrelated tyrosine kinases. The authors should confirm the important findings in Fig 4 using a more specific inhibitor of Vegfr2 (or if available, kdr or kdrl heterozygous and homozygous mutant embryos).

- We have used DMH4 as an independent VEGF inhibitor. Data is presented in Supplemental Figure 5 and are qualitatively similar to previous results with SU5416.

1-9- Lines 314 to 319. The discussion (and Fig 5) introduces the concept that PI3K signaling downstream of VEGFR2 may be disrupted in sema3fb mutants. However, this statement is unclear and the case for an involvement of PI3K is weak. For example, how would this account for the increased vegfr2, dll4 and sflt1 levels seen in sema3fb mutants? Fig 5 would benefit from removal of PI3K from the illustration, as this is confusing, and a clearer case for PI3K involvement should be added to the discussion (or this section removed altogether).

- We agree, this is not supported by our current data. Although it would be interesting for a future direction, involvement of PI3K is speculative. We have removed the PI3K discussion and removed PI3K from the figure.

Reviewer #2: The manuscript by Watterston et al focuses on the role of Semaphorin 3fb in vascular development in zebrafish embryos. The authors have made zebrafish mutants in sema3fa and sema3fb genes. Sema3fb (and not sema3fa) show vascular sprouting defects and misregulated expression of genes associated with VEGF signaling. Remarkably, reduced ISA sprouting is rescued by low dose treatment of VEGF chemical inhibitor. The authors conclude that Sema3fb functions by modulating VEGF pathway in angiogenic sprouting.

Overall, the study is well performed, the images are clear, the results are novel and describe a new role for a zebrafish Sema3F homolog in promoting angiogenesis. However, the analysis of the phenotype is mostly descriptive and leaves many questions unanswered, such as, how loss of Sema3fb expression results in increased kdrl and flt1.

Specific points:

2-1. There are multiple references to the doctoral dissertation by Halabi R. For example, data regarding the sema3fb mutant generation (which specific nucleotides are deleted in the gene), or the presence of cardiac defect are not shown and the reference by Halabi R is cited instead. It is not sufficient to cite data reported in the doctoral dissertation because it is not considered a peer-reviewed publication. Any data that is relevant to the manuscript and has not been published in a peer-reviewed journal should be included in this manuscript so that it can be reviewed.

- Although a thesis is allowed to be cited according to PLOS Genetics guidelines (it is considered a publication and all theses are publicly available), we agree this is not optimal. The pandemic has tremendously slowed publication of all data from both our labs due to reduced access to the lab continuing to this day (about 50% personnel currently, but previously 20%). Both graduate students have also left the lab and the experiments in the revision were conducted by the PI. The manuscript deriving from the cited thesis is currently in BioRXiv and is under review at PLOS Genetics. We attach the manuscript here BioRXiv (doi: <https://doi.org/10.1101/2021.05.19.444704>, reference [39]) for the reviewers to assess the data. We think however, that our Supplementary data 1 should answer all morpholino queries.

2-2. Similarly, it is said that sema3fb morpholino has been validated and the dissertation by Halabi R is cited. All data for morpholino validation need to be shown in this manuscript. Does MO cause a similar inhibition of ISA sprouting and DLAV connections as observed in sema3fb mutant?

- We have commented on this in answer to reviewer 1-7 and 2-1. The requested phenotypic data is found in Supplementary Figure 1 to show that the morpholino and mutant have similar phenotypes, and no additional phenotypes are observed after MO injection into mutant.

2-3. More complete description of the phenotype observed in sema3fb mutants is needed. Do sprouting defects recover at later stages? Are there any other vascular defects observed? Are there any obvious morphological defects observed? Cardiac defect has been mentioned; please include a description. Are homozygous mutants viable?

- We have better clarified these in our revision. Sprouting defects recover at 48 hpf, now presented in (Supplemental Figure 6). There are no additional vascular defects that we have observed, however we have not looked beyond 72 hpf. The only visible additional morphological difference is highly penetrant pericardial edema present in sema3fb mutants (91% at 48 and 72 hpf) (appended manuscript by Halabi). Cardiac defects include ventricular chamber defects (defective cardiomyocyte hypertrophy), and failure to restrict the ventricular gene expression pattern to cells around the AVC border. That the edema resolves, and the fish are viable is suggestive of compensation in later development for the loss of sema3fb.

2-4. It is important to include analysis of sema3fb MO embryos alone in Suppl. Fig. 1D and E to shown that the phenotype in double inhibited embryos is the same as in sema3fb mutants / morphants alone.

- We address this point in answers 1-7 and 2- 4. The new additional data is found in Supplemental Figure 1. The phenotype is qualitatively and quantitatively similar in wildtype and mutant injected with MO, suggesting there are no additional morpholino-induced defects.

2-5. The text says that sema3fb fish show a slight reduction in both the DA and PCV width. Yet there is no statistically significant difference in PCV width between wt and sema3fb mutants (Suppl. Fig. 2G). Also, please explain how DA and PCV were measured; is it a single measurement per embryo? It says N=3 in the figure legend for Suppl. Fig. 2F and 2G; does it mean 3 embryos; there are multiple data points in the figure? Please check the reference to panel F in the figure legend.

- We apologize, data that is not significant should not have been discussed in our first manuscript. This conclusion has been removed. We have updated the Methods for DA and PCV measurements: 5 measurements along DA and PCV directly below the ISAs imaged/measured (above the yolk extension), Panel F and G show individual counts (40-50/per embryo genotype) and numbers are now in the figure legend. We have updated the panel F reference.

2-6. It would be very informative to perform fluorescent in situ (or RNA-Scope) for kdrl, dll4 and sflt1 in sema3fb mutants. qPCR data cannot show differences in spatial localization, and these transcripts are supposed to be expressed differently in tip and stalk cells. This would allow to assay which cells (tip, stalk) show differences in transcript expression.

- This is a fruitful suggestion. We have now used fluorescent HCR for kdrl, and sflt1 to complement the qPCR in the first version. This is presented in Figure 4. We find that the overlapping expression of both transcripts in developing ISA sprouts and along the DA. We quantified the average pixel intensity within the ISAs and DA and found a significant increase in expression of both transcripts in mutant vessels (Figure 4 B and C,) which supports the upregulation previously seen with qPCR data and kdrl transgene fluorescence.

2-7. SU5416 is not specific to vegfr2 and can inhibit other tyrosine kinase receptors including vegfr3 / flt4. It would be beneficial to confirm the SU5416 rescue experiment using genetic approaches that reduce VEGF signaling. Would ISA sprouting be restored in sema3fb mutants crossed into kdrl mutant background (or kdrl morpholino can be used instead)?

- Please see the response to reviewer 1-8. We have added new data using DMH4, a second, independent VEGF inhibitor to show qualitatively similar rescue.

2-8. Because opposing results (increased kdrl and sflt1 expression) are observed it is unclear how VEGF signaling is affected in forming sprouts. Direct readout of VEGF signaling can be measured by quantifying pERK expression (from immunostaining or a live reporter line) which would help to confirm changes in VEGF signaling in sema3fb mutants.

- This was a great suggestion and we have added pERK expression. Please also see the response to 1-1.
