# Supplementary material for: Endothelial Semaphorin 3fb regulates Vegf pathway-mediated angiogenic sprouting
Source: PLoS Genet. 2021 Aug 23;17(8):e1009769. doi: 10.1371/journal.pgen.1009769 (PMC8412281; doi:10.1371/journal.pgen.1009769)
Supplement: Attachment — Submitted filename: Reply to reviewers v4.docx [file pgen.1009769.s012.docx]

**Response to reviewers, second revision.**

We thank the reviewers for their time in re-reviewing our manuscript. We have addressed these final points as outlined in blue font after each point.

Reviewer #1: The authors have addressed almost all of my comments appropriately with new experimental data or text clarifications. I have just one point that I would like clarified prior to acceptance for publication:

New data addressing the impact of sema3fb mutation on pERK levels (Fig. 4F) indicates that only homozygous null embryos exhibit an increase in pERK, whereas heterozygous embryos are unaffected. However, this is not consistent with other presented data that indicates that sema3fb is haploinsufficient. A full discussion of this discrepancy and a potential explanation is required prior to publication.

We agree that you would expect pERK levels in heterozygotes to be similar to the mutant since they show the same growth phenotype. We now realize that the analysis of hets was underpowered as compared to mutants and wildtypes in this study as there were half as many vessels analyzed (Fig 4E). This may have skewed this data and we have removed the heterozygote data from graphs 4E and 4F.

Reviewer #2: In the revision authors have satisfactorily addressed all of my major concerns. There are only a few minor issues that still need to be addressed.

1. It would be very helpful to refer to specific panels when referencing supplemental figures. Currently in most cases the reference is made to the entire figure (such as S2 Fig.) without referencing individual panels.

Sorry, this is PLOS Genetics format is in the instructions to authors. Apparently, not labeling figure parts is necessary for hyperlinking.

2. Supplemental figures show in either gray background or no background (depending which application is used to open them) which can be distracting and makes it harder to see the text. I would strongly suggest inserting white background and saving all supplemental files as .pdf (instead of .png) to make them more compatible to different readers.

We have converted them to .tif for this version and this should now be taken care of.

3. It would be helpful to include supplemental table legends next to each table (not in a separate document).

This is fixed, thanks for pointing it out.

4. Text on page 9 says that DMH4 at low dose rescued mutant vessel length. This statement is inaccurate because addition of DMH4 in sema3fb mutants resulted in shorter sprouts than sema3fb + DMSO embryos, thus addition of DMH4 made the phenotype more pronounced. This statement should be revised.

We realize the wording is confusing here. We are trying to explain that although the sprout length is also reduced mutants, they are less affected by the drug treatment as compared to wild type. We have reworded these results as follows. Hopefully, this is now clear:

*‘Similar to SU4516 treatment, wild type vessels have decreased in length following treatment with DMH4. Interestingly, although treatment with 15 µM or 25 µM DMH4 also reduced vessel length in sema3fb mutants, ISAs were still able to sprout an average 23 µm or 35 µm further than the treated wild type vessels, at each dose respectively (S6 Fig).*

5. Test should be checked carefully for grammar and errors. There are several errors throughout the text (‘23µ’ should be ‘23 µm’ on page 9, ‘ha’ should be ‘has’ on page 11, ‘find there is a significant increase fluorescent intensity’ should be ‘find that there is a significant increase in fluorescence intensity’ on page 9).

We have corrected these errors and done a deep read for other errors. Thanks for this careful reading.

6. S6 Fig. is described in the Discussion section. However, it shows original experimental results that were not discussed previously, therefore it would be more appropriate to move it to the Results section and describe it there.

We have moved the former S6 Fig to be nested in the results, now as S3 Fig. Thanks for pointing this out.

Reviewer #3: Comments to authors:

This manuscript is conveying a “confusing message”. On the one hand the authors propose that the loss of Sema3fb augments Vegf signaling. On the other hand, the authors provide evidence for upregulation of sFlt1 (Vegf-trap), which would argue for reduced Vegf bioavailability and kdrl signaling.

We think this is one of the most interesting findings, that there are both positive and negative pathways activated. We have added these points to our discussion to highlight this complex biology.

In my view the phenotype observed upon loss of sema3fb is subtle, a slight reduction (or delay) in the expansion of developing arterial ISVs. The described phenotype is not necessarily a phenocopy of Vegf loss - or gain of function. Zebrafish embryos with increased vegfaa expression show normal aISV development and filopodia numbers on aISV tip cells (Wild et al, Nat Comms, 2017). Conversely, sflt1 gain of function results in truly shorter aISVs, that in general do not grow beyond the horizontal myoseptum. Therefore, in my opinion, the data with the R2 receptor inhibitor restoring aISV progression are puzzling. I agree with reviewer 2 that the authors should try low dose vegfaa targeting morpholino of examine vegfaa+/- or kdrl+/- heterozygous mutants instead of the pharmacology tools. In the same line, the authors could examine the impact of reducing sflt1 expression levels (using low dose flt1 targeting morpholino of flt1+/- mutant) on vascular morphology, in the sema3fb mutant. The claim that increased Vegf signaling augments sflt1 expression can be substantiated in other appropriate vegf – kdrl gain of function settings (like vhl-/- mutants). The authors may want to consider the TgBAC(flt1:YFP) reporter to study the flt1 expression patterns.

These are excellent suggestions. We agree that our phenotype highlights a more subtle change in comparison to the disruptions seen with blocking Vegf signaling. We did add DMH4 data to supplement the SU4516 inhibitor data, and we now have the HCR to show that flt1 is upregulated in mutants. Together this data demonstrates that ‘slight’ increases in Vegfr2 activity can significantly disrupt ISA growth within a short window of ISA sprout elongation. We agree that it is not a simple phenocopy of reported Vegf phenotypes, and with the possible targeting approaches to test pathway regulation, however, these methods are beyond the scope of a second revision.

The authors try to link changes in endothelial nuclear cell morphology with vessel width. It has recently been shown that endothelial R2 signaling promotes endothelial cell enlargement, resulting in aISVs with a larger lumen diameter in zebrafish embryos (Klems, Nat Comms 2020). As the authors propose increased endothelial R2 signaling in sema3fb mutants, the authors should check changes in endothelial cell shape as an explanation for changes in vessel width.

We have added this reference to our discussion, and it highlights that a significant change in nuclear size is linked to disrupted vessel morphology. However, as the stalled vessel phenotype of sema3fb mutants resolves by 48hpf, our data is not directly comparable, as the published study looks at a later timepoint. We are therefore presently unable to assess changes in lumen diameter at an equivalent developmental stage.

The authors speculate that Sema3fb may control F-actin and cell elongation through disruption of the RhoA/GTPase pathway (based on reference 30). The authors can test this in their scenario – there are appropriate small Rho-GTPase inhibitors that can be used in zebrafish.

This is an excellent suggestion. However, it is beyond the scope of a second revision, but we may test this in the future.
